# Seeding Biochemistry on Other Worlds: Enceladus as a Case Study

**DOI:** 10.1089/ast.2019.2197

**Published:** 2021-02-04

**Authors:** Harrison B. Smith, Alexa Drew, John F. Malloy, Sara Imari Walker

**Affiliations:** ^1^ School of Earth and Space Exploration, Arizona State University, Tempe, Arizona, USA.; ^2^ ASU-SFI Center for Biosocial Complex Systems, Arizona State University, Tempe, Arizona, USA.; ^3^ Beyond Center for Fundamental Concepts in Science, Arizona State University, Tempe, Arizona, USA.; ^4^ Santa Fe Institute, Santa Fe, New Mexico, USA.

**Keywords:** Biochemical networks, Enceladus, Habitability, Life as a planetary process, Metabolic networks, Panspermia, Planetary protection

## Abstract

The Solar System is becoming increasingly accessible to exploration by robotic missions to search for life. However, astrobiologists currently lack well-defined frameworks to quantitatively assess the chemical space accessible to life in these alien environments. Such frameworks will be critical for developing concrete predictions needed for future mission planning, both to determine the potential viability of life on other worlds and to anticipate the molecular biosignatures that life could produce. Here, we describe how uniting existing methods provides a framework to study the accessibility of biochemical space across diverse planetary environments. Our approach combines observational data from planetary missions with genomic data catalogued from across Earth and analyzed using computational methods from network theory. To demonstrate this, we use 307 biochemical networks generated from genomic data collected across Earth and “seed” these networks with molecules confirmed to be present on Saturn's moon Enceladus. By expanding through known biochemical reaction space starting from these seed compounds, we are able to determine which products of Earth's biochemistry are, in principle, reachable from compounds available in the environment on Enceladus, and how this varies across different examples of life from Earth (organisms, ecosystems, planetary-scale biochemistry). While we find that none of the 307 prokaryotes analyzed meet the threshold for viability, the reaction space covered by this process can provide a map of possible targets for detection of Earth-like life on Enceladus, as well as targets for synthetic biology approaches to seed life on Enceladus. In cases where biochemistry is not viable because key compounds are missing, we identify the environmental precursors required to make it viable, thus providing a set of compounds to prioritize for detection in future planetary exploration missions aimed at assessing the ability of Enceladus to sustain Earth-like life or directed panspermia.

## 1. Introduction

Visionaries dream of terraforming planets while program officers fret over “contaminating” them (Rummel, 2001; Mancinelli, 2003; Musk, 2018; Kminek *et al.*, 2019). Prospective terraformers tend to believe that seeding another planet with life will require careful human or robotic (and usually Earth-assisted) cultivation. By contrast, planetary protection officers adopt a more conservative stance and assume a small, semi-sterilized spacecraft from Earth could spill life onto a planet, transforming it to a living world, in the same way that a small perturbation to a super cooled liquid would cause the entire volume to quickly crystallize. Both cases adopt an implicit assumption that Earth-life is viable outside the Earth.

However, this need not necessarily be the case, particularly given how the biochemistry of life on Earth appears so intimately coupled to its geochemistry (Shock and Boyd, 2015). In the words of Morowitz *et al.* (2008, p. 8), “the metabolic character of life is a planetary phenomenon, no less than the atmosphere, hydrosphere, or geosphere.” If this “metabolic character of life” is truly a planetary phenomenon, does this imply that life is inextricable from the planetary processes through which it emerged? Or is it possible that an infinitesimal component of our biosphere—a sliver of a sliver of Earth's biochemical diversity captured in a few species—could be enough to imbue another world with Earth's vitality?

So far, such questions have been addressable primarily philosophically, but not scientifically. To address them scientifically, astrobiology needs tools that allow testing the viability of life in diverse planetary environments and identifying what engineering modifications would be necessary to make an alien environment viable for Earth-life. Current approaches include growing specific organisms under extreme conditions (McKay *et al.*, 2012; Nicholson *et al.*, 2012; Nuding *et al.*, 2017; Heinz *et al.*, 2018; Taubner *et al.*, 2018; Stevens *et al.*, 2019) and analyzing environmental samples from planetary analog sites on Earth (Vishnivetskaya *et al.*, 2003; Amils *et al.*, 2014; Goordial *et al.*, 2017). While these approaches provide valuable insights, they require time-intensive laboratory setups, making it difficult to measure the viability of more than a few strains of organisms; or conversely, they require expensive field expeditions that are constrained by the available analog sites on Earth, as well as the diversity of organisms, which happen to be present there. Theoretical computational approaches, such as ours described herein, have the advantage of being able to test hypotheses about environments not found on Earth and to efficiently screen the potential viability of any functionally annotated organisms or communities. Our methods therefore demonstrate one path forward for astrobiologists to leverage big data approaches as increasingly detailed information about Earth's biome becomes available.

In what follows we leverage network theory as a useful tool for screening the potential viability of life, seeded from Earth, to persist in alien planetary environments. We specifically focus on determining whether or not a given biochemical system (organism or ecosystem) could, in principle, produce compounds necessary for its survival from environmentally available compounds. Our method builds on network expansion algorithms used for studying life on Earth (Handorf *et al.*, 2005), where the environmentally available compounds are identifiable as a seed set used for reactions. Using network expansion, as we will show, allows assessing from a given seed set of starting materials available in a given planetary environment what life could potentially persist there. In network expansion, reactions are permitted if they are included in the catalytic repertoire of the biochemical system of interest, for example, for an organism, this will include all reactions that the organism is known to catalyze. This information can be derived from functionally annotated genomic data (see the Materials and Methods section). Seed molecules are reacted based on the biochemically available reactions, and all newly produced reaction products become seeds for the next iteration of the algorithm. Network expansion concludes once addition of the products to the seed set produces no new additional products; the reaction network at this terminal point is referred to as the scope of the initial seed set. This relatively simple algorithm thereby provides a method for exploring how much of biochemical space is potentially accessible from a given starting point. It has been used rather extensively to study both the origin and evolution of life on Earth (Ebenhöh *et al.*, 2005; Raymond and Segrè, 2006; Borenstein *et al.*, 2008; Freilich *et al.*, 2009; Goldford *et al.*, 2017), but so far has not been implemented to study the possibility of life on alien worlds. Here, we adopt network expansion to show its use for astrobiology by demonstrating how the application of network expansion techniques to diverse planetary environments can allow a screening tool to identify what compounds could potentially be produced by Earth-life seeded in alien environments. We also show how network expansion can determine what compounds are missing that could make specific biochemical systems (organisms, ecosystems) viable in those environments.

We focus on Enceladus as a case study to illustrate our approach. Enceladus is an excellent candidate for developing data-driven computational approaches for astrobiology because its ocean-derived plume of water vapor and frozen mist make the contents of its subsurface ocean readily accessible to observation. Because Enceladus has an ocean with high pH (9–11) (Waite *et al.*, 2017), we choose to focus on the viability of prokaryotic alkaliphiles that live under similar pH conditions on Earth. By “viable” we mean to be capable of producing—using network expansion—a set of target substrates identified as necessary for producing biomass from only environmentally available starting materials. This definition of viability allows efficient screening of potential organisms, allowing for follow-up study for any organisms that meet this minimal criterion. Since other environmental factors are less well constrained, and parameters such as temperature and salinity could vary substantially across locations (McKay *et al.*, 2008), we do not place any further restrictions on the organismal metabolisms that we test with our method herein but do discuss extensions of our approach that could account for increased precision in environmental conditions and organismal attributes for testing for viability.

We also use an algorithm developed to solve the inverse problem of what seeds are necessary to produce a given set of compounds (Handorf *et al.*, 2008) to identify irreducible sets of substrates that satisfy the requirements of what these alkaliphilic organisms require in their environment to be viable. These irreducible sets provide compounds to prioritize for detection in future missions to assess the ability of Enceladus to sustain Earth-like life. If they are identified as present or absent, it would aid planetary protection efforts in assessing the risk of accidental contamination from these extremophiles. More broadly, by leveraging a big data approach merging biochemical and planetary science data sets via the application of computational methods from complex systems science, our methods provide a foundation for efficient computational assessment of the potential viability of Earth-life in other geospheres.

## 2. Materials and Methods

### 2.1. Performing network expansions

As outlined in the Introduction section, the network expansion algorithm works as follows: An organism, defined here by the fixed set of reactions which it has the ability to catalyze, can catalyze a reaction only if it has access to the necessary substrates. The initial substrates, called the seed set, are the compounds available to the organism from the environment. Initially, these are the only compounds in the organism's network. The organism catalyzes all the reactions it can based on the compounds available and then adds the new compounds produced to the set of compounds available to undergo reactions. This process proceeds iteratively until the organism can produce no new compounds. The state of the organism's network when expansion ceases is referred to as the organism's scope—and it contains all the compounds that can be synthesized by an organism, plus the seed set provided by the environment.

### 2.2. Defining the networks

To run the network expansion algorithm from a seed set, we first had to define our networks. To identify the reactions and compounds present in the metabolic networks of individual organisms, we collected data from the Joint Genome Institute's Integrated Microbial Genomes and Microbiomes database (JGI IMG/m) (Markowitz *et al.*, 2011). We located all archaea and bacteria that contained metadata on environmental pH and filtered to those organisms with pH in the range of 9–11, approximately what might be expected in Enceladus's ocean (Glein *et al.*, 2015; Waite *et al.*, 2017). For our case study, we extracted data from all 28 archaea and 266 bacteria in JGI matching these criteria, plus an additional 13 methanogenic archaea identified in the above pH range from the PhyMet2 database (Burdukiewicz *et al.*, 2018), but whose pH data were absent or limited within JGI. The full list of organisms used for network expansions can be found in Supplementary Table S1. This includes organisms with metabolisms speculated to be especially relevant to Enceladus, such as methanogens and sulfur-reducing bacteria (McKay *et al.*, 2008).

**FIG. 1.**
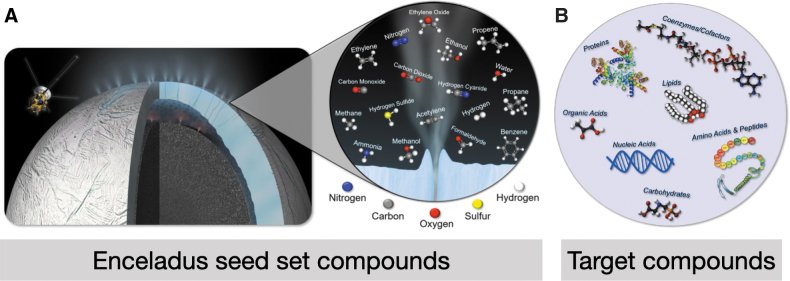
Compounds in the Enceladus seed set and biological targets for the viability of life on Enceladus. **(A)** A subset of the compounds reported to be present in the ocean of Enceladus, which are also catalogued in KEGG, a database of biochemically utilized compounds. These compounds, along with 20 others not shown, are included in our “seed set” for network expansion. We add phosphate to this seed set to test whether its presence makes a difference for the viability of diverse biochemical systems. A full list of the 38 compounds in our Enceladus seed set can be found in Supplementary Table S2 (Postberg *et al.*, 2009; Waite Jr *et al.*, 2009; Magee and Waite, 2017; Waite *et al.*, 2017; Postberg *et al.*, 2018). **(B)** An overview of the types of compounds that the target set is composed of. A listing of the specific 65 possible target compounds can be found in Supplementary Table S3 (based on the target set from Freilich *et al.*, 2009). KEGG, Kyoto Encyclopedia of Genes and Genomes. Color images are available online.

**FIG. 3.**
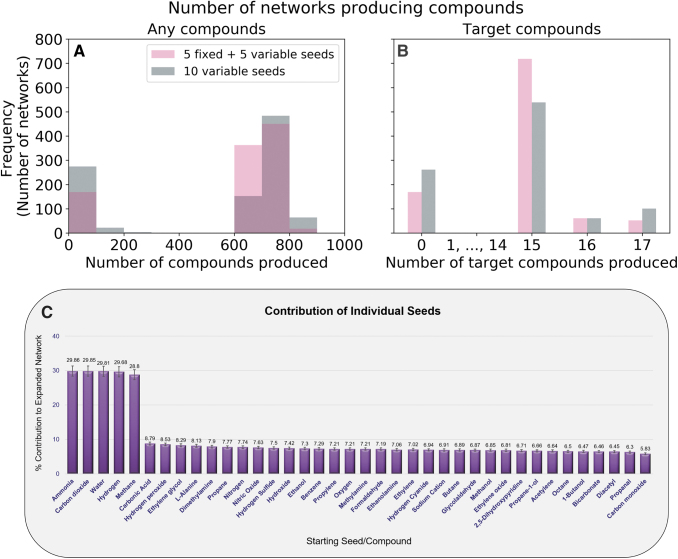
Properties of KEGG networks expanded using subsets of the Enceladus seed set. **(A)** Distribution of sizes of network scopes, expanded from size 10 seed sets, drawn from the 38 Enceladus seed compounds. Expansions were calculated when keeping the top five most abundant compounds fixed (according to Magee and Waite, 2017), and when varying all 10 seeds, drawn from a flat distribution. The *y* axis shows the number of occurrences of expanded networks within a given bin-size (bins are 100 compounds large). **(B)** Number of target compounds produced by randomized seed sets. No randomized sets produced between 1 and 14 target compounds, and none produced the number of compounds, which are produced with the full set of 38 Enceladus seed compounds (18 target compounds). **(C)** Of the networks that expanded to include at least 500 compounds, the percentage of expanded networks containing compounds is listed. Even though sulfur compounds cannot be produced without the hydrogen sulfide seed, hydrogen sulfide is not overly represented among the most largely expanded networks (those more than 500 compounds). Color images are available online.

We used a seed set of compounds that have been identified on Enceladus from observations aboard CASSINI's Ion and Neutral Mass Spectrometer (INMS) (Waite Jr *et al.*, 2009; Magee and Waite, 2017; Waite *et al.*, 2017) and Cosmic Dust Analyzer (CDA) (Postberg *et al.*, 2009, 2018). We additionally ran this seed set when including phosphate, which is likely present in small amounts from water–rock interactions, despite the lack of detection from Cassini (Guzman *et al.*, 2019). The full list of seed compounds can be found in Supplementary Table S2 (or the *x* axis of Fig. 3C), and a subset are shown in Fig. 1A.

We downloaded the Enzyme Commission (EC) numbers associated with each genome from each organism's list of “Protein coding genes with enzymes.” Each organism's list of EC numbers was mapped to the reactions, which they catalyze using the Kyoto Encyclopedia of Genes and Genomes (KEGG) (Kanehisa and Goto, 2000; Kanehisa *et al.*, 2015; Kanehisa *et al.*, 2016). Using a combination of Biopython (Cock *et al.*, 2009), the KEGG REST API, and TogoWS (Katayama *et al.*, 2010) to collect all KEGG ENZYME, REACTION, and COMPOUND data, we created reaction-compound networks for each organism. Each organism's network contains all the reactions, which all of its catalogued enzymes can catalyze, and all of the compounds involved in those reactions.

We also ran the network expansion of KEGG in its entirety (incorporating all catalogued biochemical compounds and reactions), as a model representing the catalytic and metabolic potential of the biosphere, on the seed set of Enceladus with and without phosphate (Supplementary Fig. S1).

Note that while we found 49 compounds with KEGG identifiers in the aforementioned INMS/CDA articles (Postberg *et al.*, 2009, 2018; Waite Jr *et al.*, 2009; Magee and Waite, 2017; Waite *et al.*, 2017), only 38 of those compounds are involved in valid biochemical reactions based on the version of KEGG we are using (and other constraints such as elemental balancing and avoiding the use of reactions containing compounds with glycan identifiers). Thus, for all intents and purposes, we will talk about the maximum possible Enceladus seed set as containing only 38 compounds.

We also point out that data and metadata from JGI can be incomplete or contain errors. For example, while we use the pH field in JGI to narrow down organisms for analysis in this study, there are organisms (like those we use based on identification in the PhyMet2 database) that do not have pH information listed in JGI, or whose pH range in JGI was only an optimal growth range as opposed to a full suboptimal growth range. Similarly, our analyses share the biases of many other studies such as ours, which use data from biological and biochemical databases, such as containing organisms with missing functional gene annotations and containing organisms that catalyze reactions, which are uncatalogued in the KEGG database. Our goal was to lay out a technique that can utilize all the current data as it exists, with its flaws, and be applied in the future, to produce more accurate results as data improve.

### 2.3. Alternatives to network expansion

While there are other methods that can be used to computationally assess organismal viability—relying on some combination of integer linear programming, kinetic modeling using differential equations, elementary mode analysis, and flux balance analysis (FBA)—they require either catalytic rates that are difficult to acquire and sparsely catalogued or a curated list of stoichiometrically balanced reactions (May *et al.*, 2011). FBA is perhaps the most common method for assessing organismal viability and operates by solving for the relative fluxes of reactions needed in order for steady-state production of compounds identified necessary for organismal growth. Despite FBA requiring more constrained information and computational resources, network expansion has been shown to give near identical results for identifying compounds produced, that is, the network's scope (Kruse and Ebenhöh, 2008; May *et al.*, 2011).

### 2.4. Reaction reversibility and thermodynamics

We assume that all reactions are reversible, both because the KEGG database recommends to not trust its reaction reversibility field and because reaction directionality in nature depends on the concentrations of products and reactants, and knowledge of thermodynamic conditions, which are difficult to obtain in environments on Earth let alone on other planetary bodies. In particular, while tools are available to estimate the thermodynamics of biochemical reactions from component contribution methods, they are not reliable if the temperature is assumed to be outside ∼298.15K (Jankowski *et al.*, 2008; Flamholz *et al.*, 2012; Noor *et al.*, 2012, 2013). The majority of biochemical reactions therefore do not have accurate estimates outside the standard temperature and pressure conditions at which thermodynamic properties have been measured. Thus, they do not apply to alien environments, such as those of Enceladus. One consequence of our not including thermodynamic constraints in our analyses is that our network expansions will overestimate biochemical potential (thermodynamic constraints would introduce bottlenecks in the network where reactions would not be permitted to proceed due to their high thermodynamic cost). This does not affect our general conclusions as we find, even with this overestimation, none of the organisms we study would be viable on Enceladus.

### 2.5. Defining organismal viability

We define an organism or network to be viable if, given a set of environmental seed compounds, it has the catalytic repertoire to produce all the compounds in its network, which intersects with a predefined set of target metabolites. For this study, we adopt the list of target metabolites as defined by Freilich *et al.* (2009), which are necessary for the production of biomass (Supplementary Table S3 and summarized in Fig. 1B). In that study, the authors found that the organisms that were found to be viable, based on these target metabolites, accurately predicted the ecological compositions of known environments across many habitats and bacterial metabolisms. We note that a more nuanced definition of viability should consider a gradient of potential values that depend on the possible sets of compounds produced; however, herein our goal is to establish the basic use of this formalism for astrobiology, so we leave this to future work.

### 2.6. Identifying irreducible seed sets

We follow the algorithm described in Handorf *et al.* (2008) to create random irreducible seed sets, which attempt to minimize the likelihood of obtaining seed sets with large complex biomolecules where possible. A seed set *S* is irreducible if its scope [denoted as $\Sigma \left(S\right)$] contains the target compounds *T* and no proper subset of *S* (denoted as S′) fulfills this condition. That is, *S* is an irreducible seed set if:

$$T\subseteq \Sigma \left(S\right)and\forall S\prime \subset S:T⁄\subset \Sigma \left(S\prime \right).$$

To find irreducible seed sets for each organism, we start by creating a list of all the compounds involved in all the reactions that an organism can catalyze based on its enzymatic repertoire. Going down the list, we check if removing a substrate will cause a network expansion seeded with the remaining substrates to successfully produce all target compounds. If the removal does not impact the target compounds produced, the substrate stays removed. Else, we add it back to the list. Then, we move onto the next substrate in the list, repeating until the entire list is traversed.

In this algorithm, the order of the list affects the irreducible seed set that gets identified, so it is necessary to permute the list and repeat the algorithm to identify alternative irreducible seed sets. Here, we iterate the algorithm 100 times to identify 100 possible irreducible seed sets (See SI Fig. 2 and SI Fig. 3 for additional details on this process). For each iteration, we do not want to start with a completely randomized list for each organism, because ideally, we want to remove large complex compounds, as to be left with seed sets composed preferentially with simpler compounds that are more abiogenically plausible to find in an uninhabited environment. Previous research has shown that the scopes of single complex biochemicals tend to be reachable by sets of simpler molecules (Handorf *et al.*, 2005). Because of this, we initially order every list from largest to smallest molecular weight, but then perturb them such that heavier compounds tend to stay near the top, thus getting preferentially removed. Compounds without associated weights were added in random locations in the list. Finally, to obtain results specifically relevant to Enceladus, we fix Enceladus's compounds at the top of the lists and do not allow for their removal. In this way, each network expansion provides results of what additional compounds, on top of what already is known to exist on Enceladus, are necessary to produce an organism's target metabolites.

**FIG. 2.**
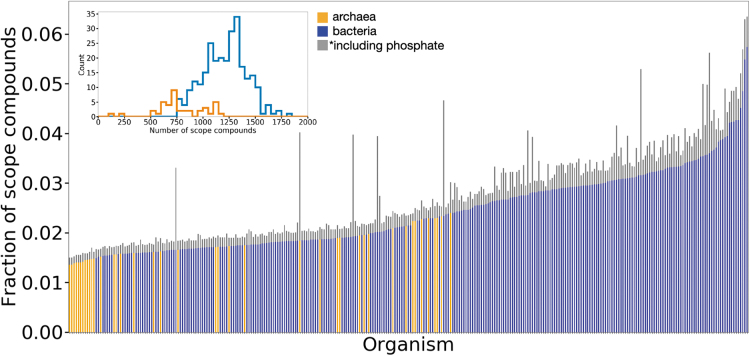
The fraction of possible scope compounds reached by the analyzed prokaryotes using the Enceladus seed set. Each bar represents an organism (orange for archaea, blue for bacteria). Bar height indicates the fraction of the maximum theoretical size of networks (if scopes were able to take advantage of full organismal reaction networks) reached using only the Enceladus seed set (A subset of which are shown in Fig. 1A). The gray addition shows how the scope size changes for all organisms when adding phosphate to the seed set. In neither case do any target compounds get produced for any organisms. The inset shows histograms for the maximum theoretical sizes of archaeal and bacterial networks, if scopes were able to take advantage of full organismal reaction networks. Color images are available online.

We again follow the method laid out by Handorf *et al.* (2008). From the list, two randomly chosen compounds with mass difference Δm get exchanged with probability *p*:

$$p=\left\{\begin{array}{cc} exp\left(-\frac{\Delta m}{\beta }\right) & if\Delta m>0\\ 1 & if\Delta m\leq 0 \end{array}\right..$$

The only exception to this rule is that if one of the compounds does not contain weight information, then p=0.5. The parameter beta represents the degree of disorder allowed in the list, where β=0 requires heavier compounds to always be above lighter ones and β=∞ ignores masses for the purpose of list ordering.

The purpose of using this equation is that it allows generating random seed sets in a way that preferentially places heavier compounds near the top of the randomized lists. This is because heavy compounds are a proxy for complex compounds and because the algorithm we use to find irreducible seed sets starts removing seeds from the beginning of the list first (leaving the more abiogenically plausible lower mass compounds in the irreducible seed sets). This algorithm ensures that when we begin to search for irreducible seed sets, our randomized lists will likely have more complex compounds removed, yet that there will be enough variation such that all randomized lists generated are not identical (which would yield identical irreducible seed sets). To allow easier comparison of our results to earlier work, we follow the choice of Handorf *et al.* (2008) and choose β=20 amu. However, we note the choice of β=20 amu is arbitrary to a degree: the most important aspect is to make the compound lists increasingly unlikely to swap high mass compounds with low mass compounds as those mass differences increase.

### 2.7. Comparing and clustering seed sets

Similarity of seed sets was calculated using the Jaccard index. Clustering was computed using scipy.cluster.hierarchy.linkage(method=“average”), where average refers to the unweighted pair group method with arithmetic mean algorithm.

## 3. Results

### 3.1. The viability of life on Enceladus: prokaryotes as a case study

Using biochemical networks generated from annotated genomic data, we show that none of the 307 tested organisms produce any target metabolites in the presence of only the limited organic and inorganic compounds currently known to be present on Enceladus. In fact, we find that the networks generated from organismal genomic data produce only a fraction of the compounds possible given their catalytic repertoire (Fig. 2, colored bars). Given how phosphorus plays a critically important role in biochemistry on Earth, this is not surprising since there is so far no direct detection of phosphorus on Enceladus. This lack of detection is possibly due to phosphorus concentration falling below Cassini instrument detection thresholds. We therefore next sought to test whether or not the lack of viability across these 307 organisms is solely due to the lack of phosphate. To do so, we tested whether adding phosphate as a seed compound would result in viable organisms. While adding phosphate does increase the scope of the organismal seed set, again, no target compounds were able to be produced (Fig. 2, gray bars). We find that to be viable, even in the presence of phosphate, these organisms additionally tend to require complex molecules and coenzymes not currently observed from Enceladus. This lack of observation of more complex/high-molecular-weight molecules could, however, be because the upper mass limit of the INMS is only 99 Da (Waite *et al.*, 2004), and the upper mass limit of the CDA is nominally 200 Da, although patterns in the observed CDA spectra indicate the presence of larger organic molecules (Postberg *et al.*, 2018).

As another end member test, we next determined whether life might be viable when the full catalytic repertoire of Earth's biosphere is available, that is, if we seeded Enceladus with a natural or synthetic ecosystem of organisms collectively containing all known enzymatic diversity catalogued in the KEGG database. This amounts to a computational experiment seeding our biosphere onto another geosphere to test their compatibility. We find, in this case, that nearly all target substrates are able to be synthesized from a seed set consisting only of the compounds currently observed on Enceladus, plus phosphate. The expansion is missing only some lipids (Phosphatidylethanolamine; 1,2-Diacyl-sn-glycerol; Hexadecanoyl-[acp]; Cardiolipin; Diglucosyldiacylglycerol; (2E)-Octadecenoyl-[acp]) and heme groups (Siroheme; Heme O) (Supplementary Fig. S1). Although the reactions in these networks are not the product of organisms that are solely alkaliphilic, our results hint that forward contamination from individual organisms may be much less concerning than contamination by a natural or synthetically engineered microbial ecosystem, which can emulate the robustness and catalytic capabilities of a large fraction of Earth's biosphere.

### 3.2. Identifying Enceladus's key environmental compounds for expanding life's biochemical networks

We also run the full KEGG network expansion using only a subset of compounds in Supplementary Table S2 as seed compounds, to observe if these smaller seed sets allow the KEGG network to expand to the same size as with all known seeds and to observe if some compounds are more important than others for expanding Earth's biochemical network on Enceladus. To do this, we generate 1000 random seed sets, each with 10 compounds. Because there are varying degrees of confidence surrounding the presence and concentrations of many of the seed compounds, we fixed the top five most abundant and confidently detected compounds (Magee and Waite, 2017). The other five compounds were randomly chosen with equally probability. Duplicate random seed sets were allowed. We find that none of the expansions reach the same scope obtained with all 38 Enceladus seed compounds, with or without phosphate. Our results are qualitatively similar even when relaxing our restriction on the five fixed compounds to allow for all the 10 compounds to come from any of the 38 Enceladus seed compounds (Fig. 3A). When we look at the relative importance of seeds for large expansions, we do not observe large differences between the seed compounds (Fig. 3C). Expansions whose seed compounds involved carbonic acid, hydrogen peroxide, and ethylene glycol are the most likely to produce networks with large scopes. Diacetyl, propanal, and carbon monoxide were the seeds least likely to lead to large scopes. However, the differences between the seeds contributing most and least are only on the order of a few percent. It is somewhat surprising that hydrogen sulfide, the only seed compound that can provide sulfur and thus allow networks to take part in reactions that use sulfur, is somewhat middling in its importance.

When we instead look at the percentage of these randomized seed sets that can produce the target metabolites, we find that the seed sets with fixed compounds more often produce target metabolites, but that the seed sets with 10 variable compounds more often produce just slightly more target metabolites (more of these networks produce 17 target metabolites than the networks with 5 fixed seeds) (Fig. 3B).

### 3.3. Identifying biochemical requirements for prokaryotic viability across diverse environments

Running network expansions on pre-established seed sets is useful for determining the set of compounds that can be part of an organism's scope if one knows the environmentally available compounds without any ambiguity. However, for the study of life on Enceladus, we do not have sufficient data to make concrete assessments of what compounds are available. We next show how network algorithms can also be used as a tool for directing our search: given a set of target molecules, it is possible to do the inverse approach and identify what seeds can produce the target set in a given reaction network. We apply this method to identify subsets of all compounds involved in a biochemical network, which could feasibly produce all the target compounds in that network.

There are two central ways to go about this: (1) identify irreducible seed sets (where no subsets of any given set can produce all target metabolites) and (2) identify the smallest irreducible seed sets (where there are no sets with fewer elements that can produce all target metabolites).

We chose to focus on the former (1): identify irreducible seed sets for the archaea and bacteria under consideration, because finding the smallest irreducible seed sets (2) is an NP-hard problem (Cottret *et al.*, 2008), and because it would result in only a single chemical environment in which a target set could be produced. The latter point is the bigger impetus for proceeding as we did—while determining the smallest irreducible seed sets would be interesting for identifying the most homogeneous (or perhaps the most simple) environments that an organism could be sustained in, it would not help us determine the breadth and distribution of additional chemical compounds necessary, on top of known planetary conditions, which could allow for functional Earth-like biochemistry across diverse planetary environments.

For the results discussed in this section, we force the irreducible seed sets to incorporate and use all identified Enceladus seeds when possible for each organism. Because of these, each organism's irreducible seed sets are specifically optimized for Enceladus.

**FIG. 4.**
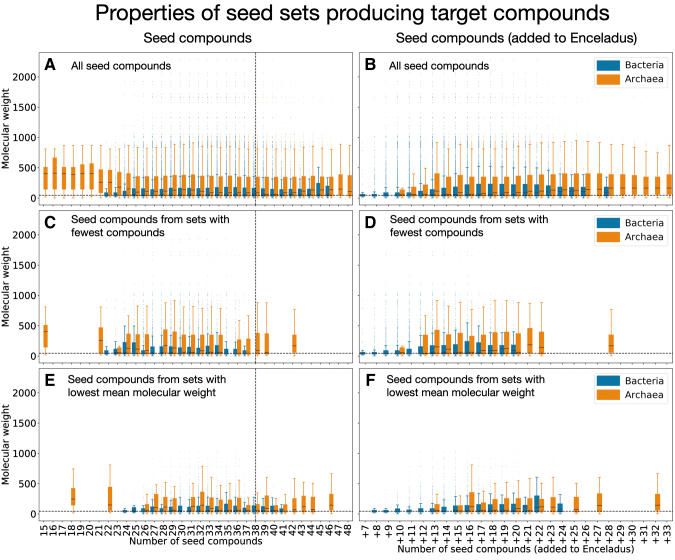
Characteristics of irreducible seed sets that produce target metabolites. Blue boxes show results of bacteria expansions, and orange boxes show properties of archaea expansions. The left column shows the molecular weights of *all seeds* in all irreducible seed sets, by the size of the seed sets they are a part of (*i.e.*, the number of compounds in the seed set). The right column also shows the molecular weights of *all seeds* in all irreducible seed sets, but by the number of seed compounds *required to be added to the Enceladus seed compounds*. The top row **(A, B)** shows distributions of molecular weights from all expansions. The middle row **(C, D)** shows the distributions of molecular weights *only from seed sets with the fewest number of compounds from each organism*. The bottom row **(E, F)** shows the distributions of molecular weights *only from seed sets with the lowest mean molecular weight from each organism*. The black dashed lines show the size and average molecular weight values for the 38 Enceladus seed set compounds. The box plot whiskers extend 1.5 times the IQR above and below the IQR. IQR, interquartile range. Color images are available online.

We find that the seed sets needed for archaeal viability are often smaller in size, but more complex (as quantified by the mean molecular weight) than the set of compounds available in the environment of Enceladus (Supplementary Table S2, Fig. 4, left column). Remember that is it possible for an organism's irreducible seed set to be smaller in size than the 38 compound Enceladus seed set, because not all organisms can take advantage of all the compounds provided by Enceladus, and thus are not forced to incorporate seed compounds that are impossible for them to use. When framing our analysis in terms of the compounds required on top of the seed compounds already available on Enceladus, we find that bacteria on average require fewer additional compounds *and* that those compounds are less complex than the compounds required to make archaea viable (Fig. 4, right column).

**FIG. 5.**
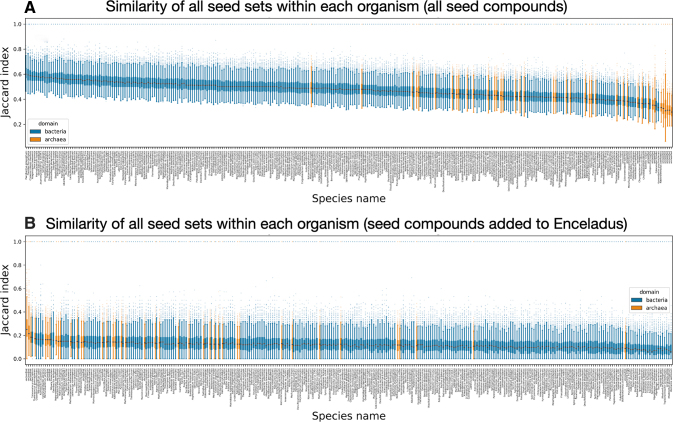
The rank ordered mean Jaccard index is shown for all 100 irreducible seed sets we calculated for each organism. Bacteria are shown in blue, and archaea are shown in orange. **(A)** Similarity is calculated using all seed compounds in all seed sets within each organism. **(B)** Similarity is calculated using only the seed compounds required to be added to the Enceladus seed compounds in all seed sets within each organism. The box plot whiskers extend 1.5 times the IQR above and below the IQR. Species names shown below each box may be difficult to read, so they are additionally provided in Supplementary Table S4. Color images are available online.

Next, we look at how similar each of the 100 irreducible seeds sets *within any single organism* is to one another. We find that across all organisms, the bacteria seed sets tend to have more self-similarity compared with the archaea (some bacteria irreducible seed sets share about 60% of their seeds) (Fig. 5A). However, when we restrict ourselves to looking at the similarity of only the compounds required to add to the Enceladus seed sets, the archaea seed sets tend to have more self-similarity compared with the bacteria (some bacterial irreducible seed sets share about 25% of added seeds required for viability) (Fig. 5B).

**FIG. 6.**
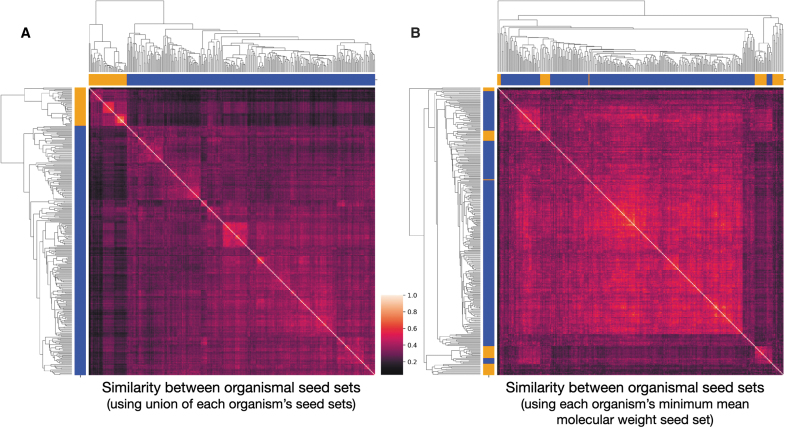
The similarity of seed sets between organisms. The clusters of two methods of organism comparisons are shown. **(A)** We take the union of all 100 seed sets within each organism and compare them with one another using the Jaccard index. **(B)** We take the irreducible seed set of the smallest mean molecular weight of all 100 seed sets within each organism and compare them with one another using the Jaccard index. In both cases, the clustering separates out the domains fairly well but is more effective when using the union of all organismal seed sets. The domain of each organism is shown as blue squares for bacteria and orange squares for archaea above and to the left of the clustermap. The scale for the heat map can be found between **(A)** and **(B)**. Color images are available online.

We then turn to examine how seed sets necessary to produce viable organisms differ *between* organisms. We find that archaea seed sets tend to be more similar to one another than bacteria seed sets. Nonetheless, comparing organisms within domains leads to similar seed sets much more often than comparing organisms across domains (Fig. 6). This result holds true even when, instead of comparing the union of seed sets of organism 1 to the union of seed sets of organism 2, we compare the minimum seed set of organism 1 with organism 2. This correlation is not as strong when looking at the irreducible seed set of each organism that has the smallest mean molecular weight (Fig. 6B). We find that clustering the Jaccard similarity between the union of organism seed sets results in more accurate clustering of the two domains we investigate (orange and blue squares above and to the left of the cluster maps show whether the row is an archaeon or bacterium, respectively). The hierarchical clustering produced from unions shows that it is possible to correctly group archaea and bacteria from only their irreducible seed sets necessary for viability. These clustering results are nearly identical even when looking only at the similarity between seeds required to be added on top of the 38 Enceladus seed compounds (data not shown).

**FIG. 7.**
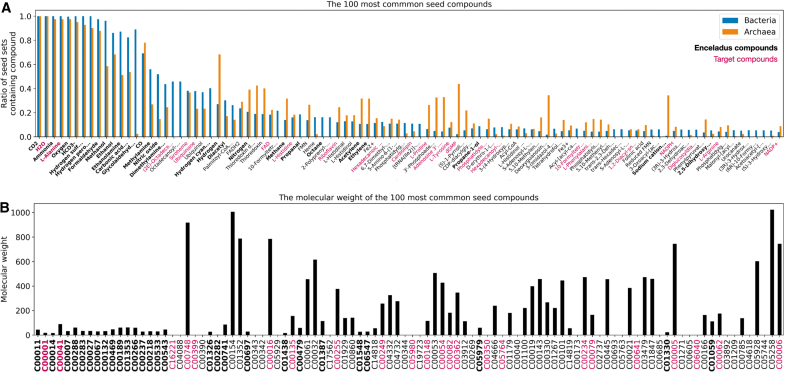
The top 100 most common seed compounds. **(A)** Rank ordered and showing the proportion of archaea (orange) and bacteria (blue) seed sets that the compounds are found in. **(B)** The molecular weights of each of the top 100 most common seed compounds. Compound names are shown in **(A)** (truncated if prohibitively long), whereas KEGG compound IDs are shown in **(B)**, in the same order. Tick labels are bold if they are part of the 38 Enceladus seed compounds and are colored raspberry if they are part of the target compound set. Color images are available online.

We turn to looking at the 100 most common seed compounds, to get some idea of the types of molecules we would expect to need to detect on Enceladus for alkaliphiles to be viable (Fig. 7). As might be expected, the majority of these compounds fall into common biochemical categories such as coenzymes, cofactors, amino acids, compounds used for fatty acid synthesis, and other key metabolic pathways. It is notable that some of these compounds are target compounds themselves (the raspberry colored tick labels), implying that these compounds are less likely to be synthesized by simpler compounds within these organismal metabolisms, and instead must be provided by the environment, or other organisms.

## 4. Discussion

It is an open question whether or not Earth-life could persist in an extraterrestrial environment, and if not, what engineering is required to make life from Earth viable in other planetary environments. Here, we have demonstrated a computational framework capable of addressing both these questions by leveraging a big data approach, merging genomic data catalogued from across Earth with planetary science data sets, adopting techniques from complex systems science. This allows us to demonstrate how these data and methods can be combined to enable efficiently screening the viability of Earth-life across alien environments. By extension, this can aid astrobiologists in identifying molecular biosignatures across planetary environments and in designing planetary protection protocols, as well as engineering synthetic or real ecosystems to survive in different planetary environments.

In particular our methods provide a screening approach for planetary protection protocols by permitting a computationally efficient means for determining whether or not a given organism, ecosystem, or synthetically engineered biosystem could survive in a given planetary environment. For example, our analysis of alkaliphiles, seeded by compounds present in the ocean of Enceladus, suggests that there is little risk of these organisms contaminating the ocean of Enceladus, as they do not have the catalytic capabilities to generate the compounds required to survive. However, forward contamination could be a much bigger risk if larger proportions of life's catalytic repertoire are transported unintentionally. At first glance, the unintentional transport of entire ecosystems from Earth rather than a few isolated species seems remarkably less likely. However, given that life often exists as ecological units and that spacecraft clean room microbial ecosystems are not well characterized (Moissl-Eichinger, 2012), the possibility of contamination by ecosystems in alien environments cannot be ruled out.

In cases where an organism or ecosystem is not viable in a certain environment because key compounds are missing, our method can identify environmental precursors required to make it viable. For example, while we find that none of the 307 prokaryotes analyzed meet the threshold for viability, the reaction space covered by this process can provide a map of possible targets for detection of Earth-like life on Enceladus, as well as targets for synthetic biology approaches to seed life on Enceladus. This includes moving beyond obvious targets such as searching for phosphate. As our analysis shows, for the alkaliphilic bacteria and archaea analyzed, just adding phosphate is not sufficient for viability; other compounds are also necessary. Our approach thus provides a set of compounds to prioritize for detection in future planetary exploration missions to assess the capacity for Enceladus to sustain Earth-like life or directed panspermia.

We also find that across different taxa, seeds are more similar between two bacteria and between two archaea than when comparing seeds between organisms from different domains, suggesting that some planetary environments could potentially host bacteria or archaea but not necessarily both. The similarity of seed sets needed for organismal viability clusters organisms into their domains, indicating that there may be further ways to identify environments suitable to specific taxonomies across planets. This is an interesting result, complementary to that of Ebenhoh *et al.* (2006), who showed that organisms that are more closely related appear to have more similar reaction scopes, as measured by the Jaccard distance. Such distinguishability in seed sets might be useful in identifying a relationship with taxonomy, for the purpose of expeditiously discerning the organisms, which could be most likely to be risks for planetary contamination or beneficial for terraformation.

Our goal in this work was to demonstrate network expansion as a screening tool for quantitative analysis of the viability of Earth-like life in alien environments. We note that this framework can naturally account for what are commonly referred to as habitability requirements, such as the presence of liquid water, as water is an essential compound for viable biosystems in our approach. Refining this approach could include exploring more metabolic diversity from Earth, studying organisms more specific to the environmental conditions on Enceladus (or other planetary bodies), analyzing ecosystem level biochemistry (*e.g.*, using metagenomic data), expanding the range of organismal pHs tested, focusing more specifically on chemotrophs or anaerobes, or adding more realistic constraints such as thermodynamic considerations. To more specifically leverage our methodology as a tool for planetary protection protocols, efforts could focus on contamination risk by analyzing candidate organisms that are known to exist in spacecraft sterilized clean rooms, such as *Bacillus pumilus* SAFR-032 (Stepanov *et al.*, 2016). A network expansion could be run on the candidate organisms' networks, with a conservative seed set, to ensure that none of the biochemistry would be viable at the spacecraft's destination. Further network expansion analyses could also be used to guide development of the composition of spacecraft materials to avoid metals, which, if in contact with certain environments, could provide rich sources of cofactors or other compounds that could support viability of Earth-life.

The subset of organisms analyzed could be expanded beyond what we include in this study to also include organisms with greater metabolic diversity, or contracted to attempt to provide a better match between what we know about organismal environments on Earth with what we know about Enceladus. With respect to determining the viability of Enceladus to support Earth's biochemistry, there are further analyses that could direct the search for compounds necessary for Earth-like life to persist. For instance, we know that the presence/absence of cofactors is a big influence on the scope size generated by a seed set (Handorf *et al.*, 2005), so prioritizing our search for these compounds would provide high scientific returns in our exploration of Enceladus or other environments. One could also measure viability as a gradient (Freilich *et al.*, 2009), allowing degrees of viability instead of an all or nothing categorization, and compare the viability of organisms in other planetary contexts with the average viability of organisms across environments on Earth. One could investigate the specific metabolic pathways that are enriched or depleted in these environments (Goldford *et al.*, 2017). Laboratory work here on Earth could also focus on better identifying reaction reversibility within organismal metabolic networks, as irreversible reaction networks would allow for more efficient algorithms used to identify irreducible seed sets (Borenstein *et al.*, 2008; Cottret *et al.*, 2008; Janga and Babu, 2008).

Biochemistry on Earth coevolved with Earth's geochemistry. Thus, there may exist significant biases in the particular compounds (in addition to environmental conditions such as T and pH not explicitly studied here) utilized by life on Earth that makes Earth-life viable only in a geochemical environment similar to the Earth's. However, a key result of our analysis is that when the catalytic capability of the entire biosphere is expanded around the Enceladus seed set (including phosphate), most of the target compounds necessary for viability are in principle producible, meaning terraformation may be possible, but would need to occur at the level of ecosystems or even planetary-scale metabolism. One strategy might be to try and produce a minimal ecosystem that can reproduce the catalytic potential of the biosphere to send to another planet. While there is much more work to be carried out to quantify the risks—or possibilities—of Earth life being viable in alien environments, the syntheses of data and methods we present lay significant groundwork for new research directions in this domain, opening a new computational space to explore the viability of biochemical networks across diverse worlds.

## Supplementary Material

Supplemental data

Supplemental data

Supplemental data

Supplemental data

Supplemental data

## Abbreviations Used

CDA: Cosmic Dust Analyzer
EC: Enzyme Commission
FBA: flux balance analysis
INMS: Ion and Neutral Mass Spectrometer
IQR: interquartile range
JGI: Joint Genome Institute
KEGG: Kyoto Encyclopedia of Genes and Genomes

## Notes

Associate Editor: Christopher McKay
