# Supplementary material for: Seeding Biochemistry on Other Worlds: Enceladus as a Case Study
Source: Astrobiology. 2021 Feb 4;21(2):177–90. doi: 10.1089/ast.2019.2197 (PMC7876360; doi:10.1089/ast.2019.2197)
Supplement: Supplemental data [file Supp_Data-Figs1-3.docx]

Supplemental information for *Seeding Biochemistry on Other Worlds: Enceladus as a Case Study*.

**
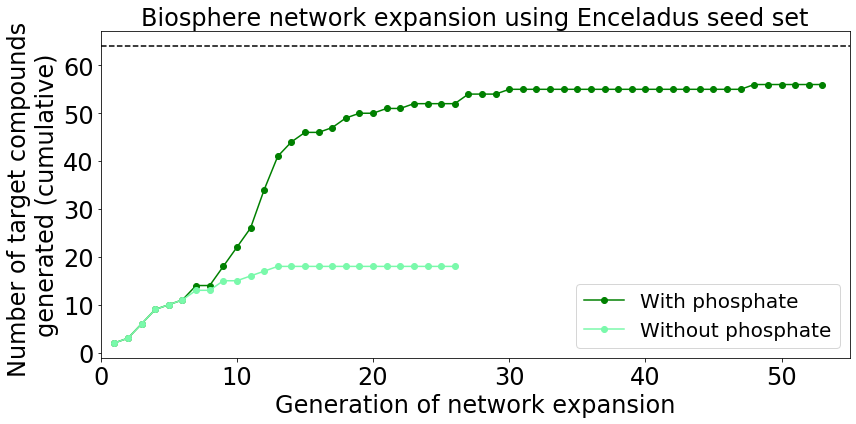
**

**SI Fig 1. Network expansion for full KEGG network with Enceladus seeds.** The network expansion of Earth’s biosphere using compounds available on Enceladus. Nearly all possible target metabolites are produced in this expansion, with some lipids (Phosphatidylethanolamine; 1,2-Diacyl-sn-glycerol; Hexadecanoyl-[acp]; Cardiolipin; Diglucosyldiacylglycerol; (2E)-Octadecenoyl-[acp]) and heme groups (Siroheme; Heme O) being absent. Without phosphate present, the KEGG expansion can only reach 18 of the 64 theoretically possible targets (and 827 compounds out of 8073 possible compounds), with 2 of those targets starting in the seed set. With phosphate, KEGG produces 56 of the 64 possible targets using the Enceladus seeds (and 3886 compounds out of 8073 possible compounds). It’s interesting that most of the targets get produced even though the KEGG network’s scope only reaches around half of all compounds in its network. Even though our new enceladus seed set is comprised of 49 compounds with identified KEGG IDs, our KEGG network only contains 38 of these Enceladus seeds (ie. with a fully expanded scope, it can only ever contain 38 of the 49 Enceladus seeds).

# Deciding to how many irreducible seed sets to calculate

Two of the most relevant features of the irreducible seed sets are: 1) their size (number of compounds), and 2) their molecular diversity. That is, ideally we would calculate *n* irreducible seed sets where *n* is enough to give us: 1) an accurate estimate of how many compounds tend to be present in any irreducible seed set, and 2) how many unique compounds exist across all seed sets for a given organism (the molecular diversity of the seed sets).

We did some simple convergence tests within and across organisms, and found that the first ~50-100 irreducible seeds we get a good idea of #1, the average number of seeds. Additionally, we find that by calculating about 100 minimal seed randomizations, we can estimate ~75% of the diversity of seeds that we'd likely see after 1000 or so minimal seed randomizations. Based on the computational time required to calculate irreducible seed sets, we made a tradeoff to calculate 100 seed sets per organism. We’re confident that we’re getting a good estimate of the average size of our irreducible seed sets, and we acknowledge that more information (with diminishing returns) could be gained about molecular diversity if we continued to calculate more irreducible seed sets.


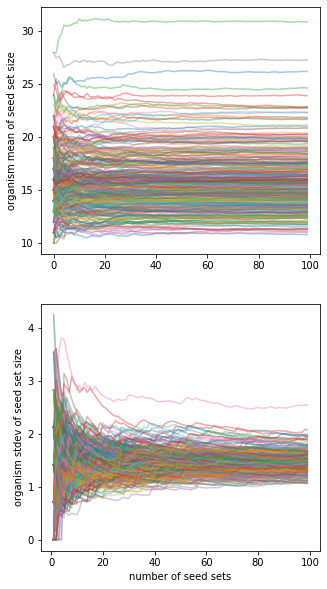

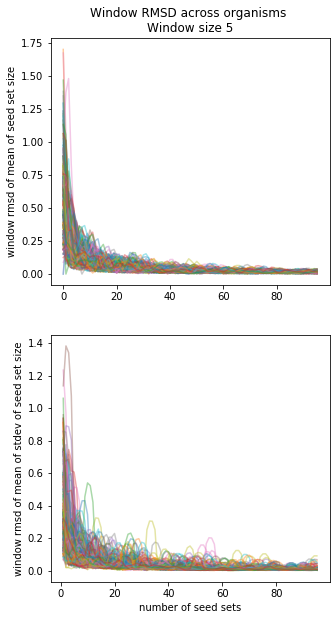


**SI Fig. 2 The average number of compounds in irreducible seed sets across organisms.** The two top plots show the mean number of compounds across all of an organism’s irreducible seed sets, as new irreducible seed sets are added to the analysis. The bottom two plots are showing the standard deviation of the mean. The left column shows raw values, which vary a lot since each organisms irreducible seed sets tend towards different sizes. To correct for this, we also calculated a windowed root mean square deviation. These calculations are shown for all organisms (each one a separate color). The x-axis increases from 0 to 100, showing how the means and standard deviations change as they account for additional irreducible seed sets. These plots show that we have good confidence in estimating the average number of compounds in irreducible seed sets for each organism after calculating >40 or so of these seed sets. These results were calculated for a window size of the previous 5 timesteps, but hold for other values as well (we also tested 3 and 8).

The calcuations we used for windowed root mean square deviation are as follows in python code:

def window_rmsd(xs):

return (np.sum([(x-np.mean(xs))**2 for x in xs])/(len(xs)-1))**.5

def sliding_window_rmsd(means,window_size):

result = []

for start in range(len(means)-(window_size)+1):

result.append(window_rmsd(means[start:(start+window_size)]))

return result

But how well do our seed sets cover the diversity of molecules possible in the irreducible seed sets?


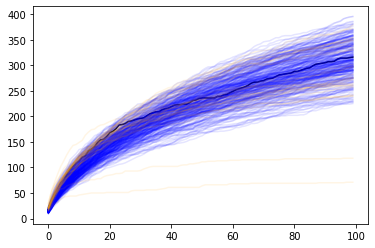

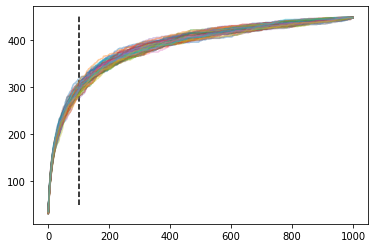


**SI Fig. 3.** **Species accumulation curves for the organisms in our study.** This plot shows “species accumulation curves” for unique compounds discovered in irreducible seeds (across organisms). The x-axis shows how many irreducible seed sets have been taken into account, and the y-axis shows the number of unique compounds discovered. Each line an accumulation curve for a single organism (blue are bacteria and orange are archaea). Here we also see that we keep discovering new compounds even up to the last calculated irreducible seed set. So, in the right plot we calculated 1000 minimal seed sets for a single middling organims (the black one in the left plot) in order to see if we can flatten our accumulation curve. The black dashed line shows 100 irreducible seeds for reference. We find that the curve does flatten out quite by 1000.

So the takeaway is, by calculating ~100 minimal seeds we get a good estimate of the average number of compounds in an irreducible seed set across organisms. We get a slightly less good estimate of the diversity of seeds across all an organism’s irreducible seed sets, finding ~75% of the diversity of seeds that we'd likely see after 1000 or so minimal seed randomizations. However, for computational tractability we made a tradeoff here to calculate only the first 100 irreducible seed sets, to balance knowledge on species accumulation with diminishing returns for calculating more irreducible seed sets.

**SI Table 1. List of taxa used in this study.** Select metadata of the organisms used in this study is listed. All data is from JGI’s IMG/m. The taxa listed include all the organisms in JGI’s database with a listed pH overlapping the range 9-11, at the time of retrieval (Sept. 2018). Additionally, our study includes organisms with a listed pH range of 9-11 identified using the PhyMet2 database. In this table, those organisms identified from PhyMet2 either have missing pH values, or have pH values outside of the aforementioned range (since the pH column is strictly from JGI).

**SI Table 2. Compounds inferred from Enceladus observations, for use in our seed set.** Values contain a 1 if they were identified in the source publication listed in the column header. The column “contains reactions” contains a 1 in rows where the associated KEGG compound is involved in any KEGG reactions (i.e., if the entry contains a 0, it means that the KEGG database does not contain any reactions which involve that compound). KEGG ID is listed as 0 for compounds which are not found in the KEGG database. The only compound listed which was not found in any source paper is phosphate (row 4).

**SI Table 3. Compounds used in our target set.** Based on Freilich et al. 2009.

**SI Table 4. Jaccard values corresponding to main text Figure 5.** The first three columns correspond to points shown in Figure 5A. The other three columns correspond to points shown in Figure 5B.
